# Transitional Cell Carcinoma within a Portion of Inguinally Herniated Bladder

**DOI:** 10.1155/2013/610312

**Published:** 2013-05-23

**Authors:** Matthew A. Uhlman, Nathan A. Bockholt, Amit Gupta

**Affiliations:** Department of Urology, University of Iowa, 200 Hawkins Dr., 3 RCP, Iowa City, IA 52242-1089, USA

## Abstract

Bladder herniation within the inguinal canal is a relatively uncommon finding. We report an even less-common occurrence of transitional cell carcinoma located within a portion of inguinally herniated bladder. Fewer than 20 reports exist in the literature describing this scenario.

## 1. Introduction

 Bladder herniation into the inguinal canal is uncommon. Previous reports have shown bladder to be present in 0.36–4% of inguinal hernias [1, 2] though some estimates have placed the rate as high as 10% in males over 50 [3, 4]. This may include true bladder or a bladder diverticulum. The vast majority of bladder hernias are asymptomatic with only small portions of bladder within the canal; however, some extend into the scrotum. These larger herniations are termed a scrotal cystocele and may present with two-stage voiding, decrease in scrotal size after voiding [5], or increased urinary flow by hernia compression or scrotal elevation (Mery's sign) [6]. Numerous other reports exist about inguinoscrotal bladder hernias that presented with symptoms including lower urinary tract symptoms, renal failure, and gross hematuria [4–6]. 

 The presence of bladder carcinoma within a herniated portion of bladder is exceedingly rare, with less than 20 reports in the literature over the past 50 years [6–8]. Treatment is dictated by pathology, presence and amount of tumor elsewhere in the bladder, and depth of invasion. 

## 2. Case Presentation

 A 90-year-old man was seen in consultation for complaints of acute onset of gross hematuria. The patient reported that for roughly one month he had intermittent “tomato juice” colored urine. He had no previous reports of urologic malignancy or hematuria and denied any weight loss, lower urinary tract symptoms, or irritative voiding symptoms. Upon consultation, urinalysis was positive for blood, and a review of urine labs performed in the previous three months demonstrated microscopic hematuria. Physical examination revealed bilateral inguinal hernias, the right larger than the left, but neither causing symptoms. All pertinent biochemical labs were within normal limits.

**Figure 1:**
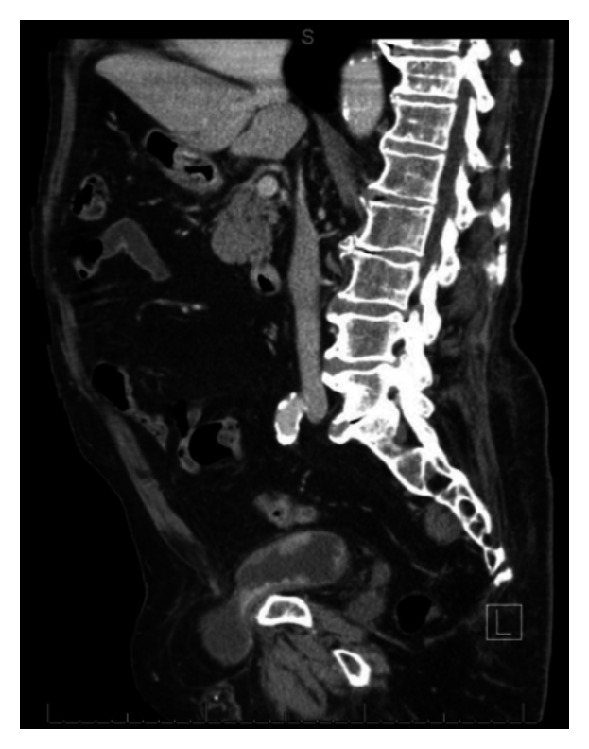
Sagittal view of herniated bladder with transitional cell carcinoma.

**Figure 2:**
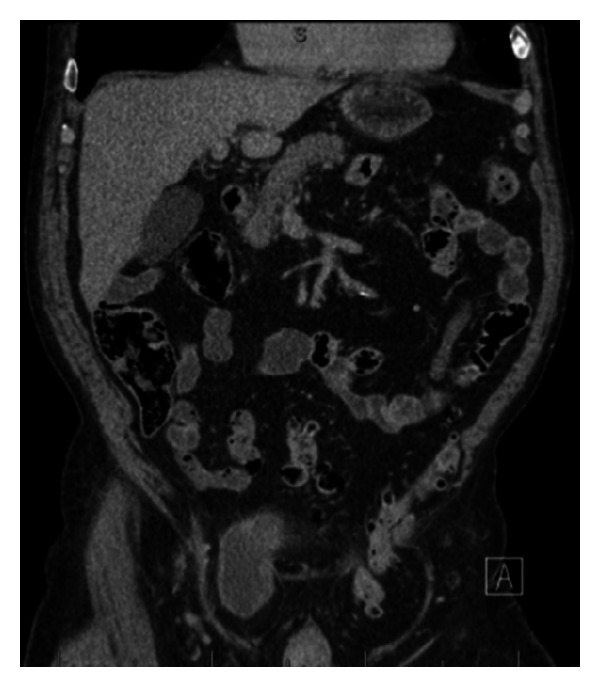
Coronal view of herniated bladder containing transitional cell carcinoma.

**Figure 3:**
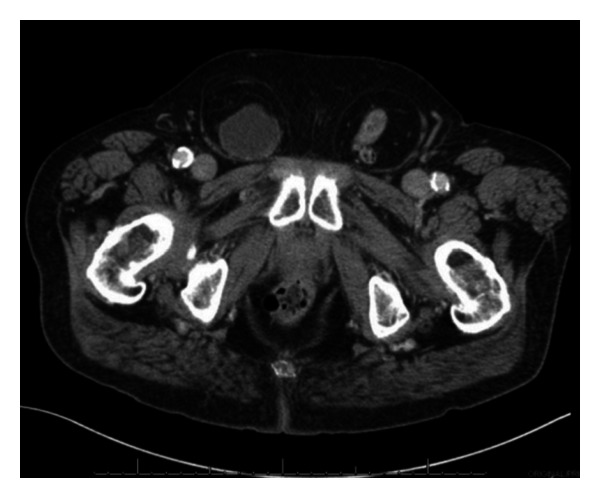
Axial view of bladder hernia in right inguinal canal and colon in left inguinal canal.

CT urogram revealed a number of bladder masses suspicious for malignancy. A large right-sided bladder herniation extended down into the right inguinal canal and scrotal sac, consistent with physical exam findings. Due to patient positioning, this herniated portion did not contain any contrast on delayed phases of CT; however, wall thickening was observed and a mass was noted in the neck portion of the diverticulum that extended through the inguinal canal (Figures 1, 2, and 3). Additionally, a left-sided hernia was noted to contain descending and proximal sigmoid colon. Cystoscopy revealed a number of large masses within the bladder, one of which emanated from the entrance into the herniation. Due to the acute angle necessary to enter the herniation, the inside was not visualized. Bladder washings obtained during cystoscopy revealed high-grade carcinoma. Two weeks later, the patient underwent a transurethral resection of bladder tumor. During the surgery, the herniation was reduced; however, the tumor burden was too large to be resected completely. Notably, the bladder was mobile on bimanual exam at the end of the case. Pathology returned as T1 high-grade transitional cell carcinoma. Muscle was present in the specimen. The patient was counseled on possible surgical, chemotherapy, and radiotherapy options and declined all. He is currently doing well and remains asymptomatic.

## 3. Discussion

 The presence of carcinoma within a herniated portion of bladder can present unique therapeutic challenges. Given that most bladder hernias are asymptomatic, the majority are discovered at the time of surgery [2]. If suspected, the best imaging modality for detecting a bladder hernia and the contents within is a CT Urogram, with recent suggestions that 3-D CT scans may offer further advantages for surgical planning [9].

 Ideally, including the current case, herniations are reducible at the time of cystoscopy, providing means for adequate transurethral resection of tumor and future treatments. Care must be taken to avoid perforation, especially if a bladder diverticulum is suspected on radiological imaging. 

 Few reports have discussed the management of such cases though a small number of them have described bladder sparing approaches with the removal of the TCC—containing portion of bladder [6, 10–12]. Three of these cases were for true bladder containing hernias, while the others were for hernias that contained bladder diverticula. The incidence of primary carcinoma developing within a diverticulum is estimated at 1–10% [13, 14], accounting for roughly 1.5% of bladder cancer [15]. While the exact rate of transitional cell carcinoma within a herniated portion of bladder is unknown, it is no doubt very small, with this report being roughly the 20th to date.

## 4. Conclusion

 Bladder herniation and scrotal cystocele are rare entities. Cancer contained within these findings is even more uncommon and treatment presents a unique challenge. Here we describe a patient who presented with gross hematuria and was found to have transitional cell carcinoma contained within an inguinally herniated portion of bladder. Pathologic diagnosis is crucial in determining the proper course of treatment and reduction of the herniation may provide visualization for adequate transurethral resection and future intravesical treatments.
